# A Microfluidics Approach for Ovarian Cancer Immune Monitoring in an Outpatient Setting

**DOI:** 10.3390/cells13010007

**Published:** 2023-12-20

**Authors:** Sarah Libbrecht, Ann Vankerckhoven, Koen de Wijs, Thaïs Baert, Gitte Thirion, Katja Vandenbrande, Toon Van Gorp, Dirk Timmerman, An Coosemans, Liesbet Lagae

**Affiliations:** 1 Life Science Technologies, imec, B-3001 Leuven, Belgium; sarah.libbrecht@imec.be (S.L.);; 2 Department of Oncology, Laboratory for Tumor Immunology and Immunotherapy, Leuven Cancer Institute, KU Leuven, B-3000 Leuven, Belgium; ann.vankerckhoven@hotmail.com (A.V.); an.coosemans@kuleuven.be (A.C.); 3 Department of Gynecology and Obstetrics, UZ Leuven, B-3000 Leuven, Belgium; 4 Department of Oncology, Gynecological Oncology, KU Leuven, B-3000 Leuven, Belgium; 5 Department of Development and Regeneration, KU Leuven, B-3000 Leuven, Belgium; 6 Physics Department, KU Leuven, B-3000 Leuven, Belgium

**Keywords:** flow cytometry, lab on chip, immune monitoring, ovarian cancer, immunotherapy

## Abstract

Among cancer diagnoses in women, ovarian cancer has the fifth-highest mortality rate. Current treatments are unsatisfactory, and new therapies are highly needed. Immunotherapies show great promise but have not reached their full potential in ovarian cancer patients. Implementation of an immune readout could offer better guidance and development of immunotherapies. However, immune profiling is often performed using a flow cytometer, which is bulky, complex, and expensive. This equipment is centralized and operated by highly trained personnel, making it cumbersome and time-consuming. We aim to develop a disposable microfluidic chip capable of performing an immune readout with the sensitivity needed to guide diagnostic decision making as close as possible to the patient. As a proof of concept of the fluidics module of this concept, acquisition of a limited immune panel based on CD45, CD8, programmed cell death protein 1 (PD1), and a live/dead marker was compared to a conventional flow cytometer (BD FACSymphony). Based on a dataset of peripheral blood mononuclear cells of 15 patients with ovarian cancer across different stages of treatment, we obtained a 99% correlation coefficient for the detection of CD8+PD1+ T cells relative to the total amount of CD45+ white blood cells. Upon further system development comprising further miniaturization of optics, this microfluidics chip could enable immune monitoring in an outpatient setting, facilitating rapid acquisition of data without the need for highly trained staff.

## 1. Introduction

Ovarian cancer is the fifth-most lethal cancer type for women [1]. High-grade serious ovarian cancer (HGSOC) is the most common subtype, with five-year survival rates as low as 30% [2]. This is in part due to the paucity of early onset symptoms and the lack of diagnostic specific markers causing most cases to be diagnosed in advanced disease stages. Around 82% of women with HGSOC are diagnosed after the cancer has extensively spread throughout the abdominal cavity or beyond (stage III and IV according to the International Federation of Gynecology and Obstetrics (FIGO) classification [3,4]).

The introduction of immunotherapies as anticancer treatments caused renewed enthusiasm as they have shown great successes in other cancer types like melanoma [4]. More recently, a combination of different checkpoint inhibitors showed promising results where the median overall survival was not yet reached after a follow up of 60 months [5]. Following these successes, many immunotherapies were also tested in other cancer types, including ovarian cancer. Unfortunately, the immunologic situation in ovarian cancer seems to be more complex. Early clinical trials with immunotherapy resulted in low response rates [6,7]. This discordance could partially be explained by the fact that clinical trials have almost no biomarkers implemented to guide therapy management but only rely on biopsies taken prior to the primary treatment. Current knowledge, however, clearly states that each metastatic spot in ovarian cancer has its own unique tumor immune microenvironment [7,8,9]. In addition, multiple groups, including our own, have shown that the composition of the immune system changes throughout disease progression and after introducing different therapies, making the design of (immuno)therapeutic strategies based upon biopsies taken only at the beginning of treatment a risky business [10,11]. Monitoring immune changes at a systemic level, for example, via flow cytometry, would provide insight into tumor–immune dynamics and how they change during treatment. Furthermore, after clinical implementation of immunotherapies, one could adjust the therapeutic strategy throughout the disease/treatment course and tailor it to patients [12]. However, only a minority of clinical trials implement immune profiling during the treatment with immunotherapies since the current flow cytometry systems are bulky, complex to handle, and expensive. In addition, most cytometry systems are centralized in specialized lab facilities, as they need to be operated by qualified personnel, making immune profiling logistically cumbersome to implement in clinical practice.

Microfluidics chip-based flow cytometry is a new, rapidly evolving technique that offers a solution to some challenges experienced in conventional flow cytometry [13]. Readouts can be multiplexed to allow for fast readout while maintaining a portable format. Furthermore, these systems can be fully enclosed and developed as disposables for a specific panel, eliminating the risk of cross-sample contamination. These features could facilitate fast diagnosis and alterations of treatment strategies based on longitudinal patient-specific data and eliminate the need for cryopreservation or cumbersome sample logistics to centralized laboratories. Nevertheless, the development of these systems for clinically relevant assays has two major hurdles that need to be overcome: fluidic throughput and optical multiplexing in a miniaturized format. The optical challenge entails both the development of miniaturized or integrated systems to guide light onto multiple channels and the detection of light emitted and/or scattered by the particles. Both our groups and others have developed low-loss integrated waveguides in silicon that facilitate compact and alignment-free illumination of particles in fluidic channels [14,15,16,17]. For the detection of the emitted light, there have been reports that show that integrated detection of multiple fluorescent markers is possible in various degrees ranging from integrated lenses [18] to full collection of light through grating couplers and waveguides, integrated on the microfluidic chip [13,19,20]. Considering the fluidic throughput challenge, multiple design concepts and platforms are suited for flow cytometry applications on chips, but not all are suited for processing sufficient cells in a short time. We believe that the only way to achieve a high throughput by harvesting microfluidics parallelization and miniaturization of optics is by using an integrated silicon platform. Although incorporation of these features will increase the price of the disposable, we believe that the induced cost-reduction of manpower, logistics, maintenance, and training will compensate for that.

While our earlier work focused on pushing the limitation in speed and integrated detection [14,17], this work focuses on the fluidic principle that can allow high-throughput fluidic parallelization on a silicon platform, enabling full integration. The combination of both will allow for the evolution of a bedside tool. In this report, we seek to compare the analysis of an immune readout on a conventional cytometer (BD FACSymphony) to our newly developed microfluidics-based cytometry chip. For this proof-of-concept study, we focused on the detection of the programmed cell death receptor 1 (PD-1) on T cells. The ligand for this receptor is expressed on ovarian tumor cells and shown to induce T cell apoptosis. Binding of the ligand to the PD-1 receptor favors the immune response escape of these tumor cells. Therefore, inhibitors for PD-1 have drawn a lot of attention in the field of immune-based therapy development [21].

For our study we have evaluated the detection of PD-1 on T cells using a basic four-color panel (CD45, CD8, PD1, and a live/dead marker) in a small dataset of 15 patients with ovarian cancer across different stages of treatment. Within this group, we were able to show that a high correlation can be achieved with our new method, allowing for the detection of biological trends that is similar to that achieved with a conventional flow cytometer.

## 2. Materials and Methods

### 2.1. Study Design

**Figure 1:**
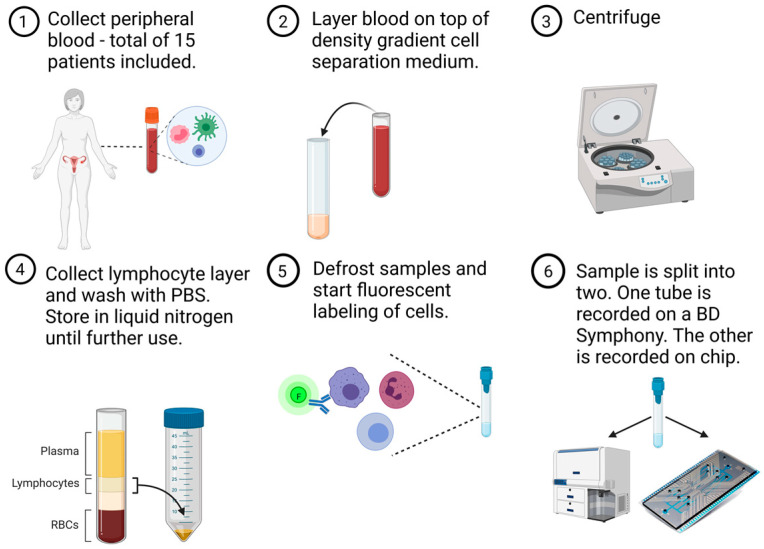
Overview of experimental study design in a six-step process. Peripheral blood samples were obtained from 15 patients with ovarian cancer. White blood cells were isolated by means of a density gradient centrifugation and frozen until further use. Batches of four to five samples were defrosted and labelled with fluorescent dyes. Each fluorescently stained patient sample was split into two equal parts to perform simultaneous but separate acquisition on a conventional flow cytometer (BD FACSymphony) and our own, silicon, microfluidics-based chip cytometer (Figure created in BioRender).

For this proof-of-concept study, we prospectively collected peripheral immune cells from 15 patients diagnosed with high-grade serous ovarian cancer (HGSOC) at various stages during their disease course. Full details on age, stage, moment of blood sampling, and disease course of the patients can be found in Table 1. Samples were taken between November 2020 and August 2021. Patients with known (auto)immune diseases or receiving immune-modulating drugs were excluded from this study. All patients granted written, informed consent. This study was approved by the local ethics committee (The Ethics Committee Research UZ/KU Leuven (EC Research)) (s63209) at the University Hospitals Leuven (Belgium). A schematic representation of the experimental design can be found in Figure 1.

### 2.2. Sample Preparation

Whole blood samples were obtained at indicated time points using Vacuette NH sodium heparin tubes (ref 455051, BD Biosciences, Erembodegem, Belgium). Next, peripheral blood mononuclear cells (PBMCs) were isolated using Lymphoprep Density Gradient Medium. Isolated cells were counted, frozen to −80 °C using a slow-freeze protocol (max −1 °C/min), and subsequently stored in liquid nitrogen until analysis. PBMC samples were defrosted in batches. Per batch, samples from four to five patients were simultaneously defrosted and prepared for analysis. Upon defrosting, cells were washed with 10 mL of ice-cold Dulbecco’s Phosphate Buffered Saline (DPBS) and centrifuged for 10 min at 4 °C at 400 RCF. Next, cells were counted with an automated cell counter (ABX Micros 60, Axonlab, Zaltbommel, The Netherlands) and resuspended to one million cells per 100 µL for staining. To exclude dead cells, samples were stained with a viability dye (PE-Texas Red, REF 32006-T, Biotum, Fremont, CA, USA) and incubated for 20 min at 4 °C in the dark. Cells were washed before adding the following cell surface marker stains: CD45 (PE-Cy5, clone HI30, REF 15-0459-42, eBioscence, Merelbeke, Belgium), CD8 (PE-Cy7, clone HTT8, REF 300914, Biolegend, Amsterdam, The Netherlands), PD1 (PE, clone REA1165, REF 130-120-388, Miltenyi, Gladback, Duitsland). All dye concentrations were optimized via prior titration experiments. A fluorescence minus one (FMO) control for PD1 positivity was included for all patient samples. After washing, cells were fixed with 4% paraformaldehyde for 20 min at 4 °C. Next, cells were washed again and resuspended thoroughly with 1% bovine serum albumin (BSA)-DPBS before dividing each sample into two equal parts for separate but simultaneous acquisition on both measurement systems. Before sample acquisition on chip or by the conventional cytometer, the sample was filtered through a 30 µm mesh filter. Both sample acquisition as well as sample analysis were performed by separate and double-blinded researchers (AVK and SL).

### 2.3. Conventional Flow Cytometry Measurement

Samples were acquired on the conventional flow cytometer BD FACSymphony (BD Biosciences, Erembodegem, Belgium). For these experiments, only the 561 nm yellow-green laser of the instrument was used. The system runs on BD FACSDiva software (Version 8.0.01, BD Biosciences, Erembodegem, Belgium). Specifications on detector configurations can be found in Supplementary Table S1. Analysis of raw data was performed with FlowJo Software (Version 10.7.1, BD Biosciences, Erembodegem, Belgium). See Section 2.5 for the gating strategy.

### 2.4. Microfluidic Chip and Cytometry Measurement

**Figure 2:**
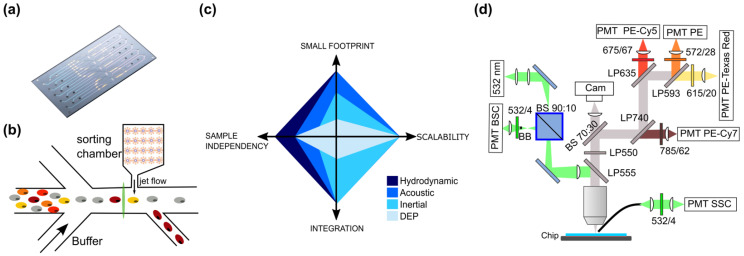
Description of the chip cytometer setup. (**a**) Example photograph of the chip design that contains 5 fluidically independent microfluidic channels. (**b**) Illustration of one microfluidics channel. Buffer fluids were used that act as sheath fluid to hydrodynamically focus cells into the center of the laser beam (green vertical line). A sorting chamber was present but not used in current study (**c**). Diagram of the key features of different fluidic focusing principles. The relative score of the different concepts for footprint, scalability, ease of integration, and sample dependency were provided by the size of the color diamond in the axis of the property. The higher the concept scores on a property, the closer the diamond will extend to the maximum of the corresponding axis. Relative scores between the hydrodynamic, acoustic, inertial, and DEP concept were provided based on the results reported in the literature [22,23,24,25,26,28,29,30,31,32,33,34,35]. (**d**) Schematic of the optical system.

Microfluidic cytometry and sorting chips have been developed by imec and are based on a microfluidic switching principle that is mild to cells. It uses integrated microheaters in a sorting chamber to create local vapor bubbles, a principle that is similar to that employed in inkjet printers. These bubbles induce a pressure and a force that pushes cells towards a side outlet. The bubble jet sorter chips used in this study were fabricated at imec on 200 mm silicon wafers based on the first generation described by De Wijs et al. [22]. In this generation of the device used in this study, the fluidic channels were fabricated in silicon instead of an adhesive. The use of silicon channel walls and a quartz cover glass minimizes autofluorescence background signals. Furthermore, it allows the process to be easily transferred to mass manufacturing. As depicted in Figure 2a, one microfluidic chip contains five microfluidic channels that can be fluidically addressed separately. The layout of one microfluidic channel is shown in Figure 2b.

To fluidically focus the sample in a single-cell sample stream on a silicon platform, multiple fluidic principles can be used, such as DEP [23,24,25], acoustic focusing [26,27,28,29,30,31], inertial focusing [32], and hydrodynamic focusing [22,33]. As can be seen from Figure 2c, hydrodynamic focusing scores better in the four properties we were looking for: implementation on a small footprint, easy to integrate, scalable to multiple channels, and independent to the size and nature of the cells in the sample (Figure 2c). Other concepts, such as inertial focusing, for example, also scored high on scalability, as shown by Zuhkov et al. [34], but required more space and were dependent on the cell sizes available in the sample. Once the cells are focused into the main channel, using 2D hydrodynamic focusing, they pass a narrow interrogation point downstream. Cells can exit the chip either via the main channel or, in case the sorting chamber is activated, a side channel.

In this study, we validated the cytometric aspect of this microfluidic chip. For this purpose, an optical system was constructed based on commercially available optical components to detect viable CD45+CD8+PD1+ PBMC based on both scatter characteristics and fluorescence (Figure 2d). To visualize the fluorescence of passing cells, a 532 nm continuous wave laser (OBIS 532 LS FP, 40 mW, Coherent, Gent, Belgium) was focused through a 20×/0.42 NA (Mitutoyo, Beveren, Belgium) lens objective in the center of the 120 µm-wide main channel. Beam shaping was performed to obtain a beam width of 5 µm. Backscattered light was collected by the objective lens and reflected by a dichroic mirror (DM LP550) to a PMT detector (BSC, H10723-01, Hamamatsu, Mont-Saint-Guibert, Belgium). A beam blocker (BB) was placed in the back focal plane to block reflected laser light and scattering from the fluidic channel walls. The collected fluorescence light was split by a beam splitter (BS 70:30) towards a CCD camera (Axiocam 503, Zeiss, Oberkochen, Germany) for online monitoring of the sorting process and towards four fluorescence PMT detectors (Fl1-FL4, H10723-01, Hamamatsu, Mont-Saint-Guibert, Belgium) for cell detection. An optical path composed of several dichroic mirrors and bandpass filters were used, as depicted in the scheme, to allow detection of PE, PE-Texas Red, PE-Cy5, and PE-Cy7. The side scatter signal (SSC) was detected on a PMT (H10723-20, Hamamatsu, Mont-Saint-Guibert, Belgium) through a multimode optical fiber with a 1000 µm core (M56L02, Thorlabs, Bergkirchen, Germany). Optical signals were processed on an FPGA with integrated ADC (USB-7855R, National Instruments, Austin, TX, USA) at a sample frequency of one million samples per second and processed by custom software (Labview, version 2014 SP1, National Instruments, Austin, TX, USA). Peak detection was set to the backscatter signal. Based on the signals measured, the same metrics as in conventional flow cytometry were derived for analysis: height and area of the signal peak. The data were stored in a format that could be read in FlowJo software (Version 10.7.1, BD Biosciences, Erembodegem, Belgium) for further processing. See Section 2.5 for the gating strategy.

### 2.5. Data Analysis and Gating Principle

The obtained data were analyzed using FlowJo software. For every patient, different subpopulations were identified based on a standard flow cytometry gating strategy. Like in conventional flow cytometry, on both systems, we excluded debris and doublets based on forward and sideward scatter information. Next, dead cells were excluded based on viability staining. Based on this subpopulation, we selected the total CD45+ population (versus side scatter) and subsequently selected all CD45+PD1+ cells based on FMO controls. From the same viable CD45+ population, we also gated all CD45+CD8+ versus CD45+CD8− cells. Both cell populations were subsequently gated for their PD1+ cells based on FMO controls. An example of this gating strategy on a representative sample can be found in Supplementary Figures S1 and S2 for both the chip and conventional cytometer, respectively.

### 2.6. Statistics

Absolute cell numbers of CD45+ cells and percentages of cell populations can be found in Supplementary Table S2. Statistical analysis was performed in Origin 2019. To investigate the agreement between the measurements obtained on the conventional cytometer (BD FACSymphony, BD Biosciences, Erembodegem, Belgium) and the microfluidic cytometer chip, we performed linear regression analysis and Bland–Altman analysis. For the former, the data obtained on both systems for each patient were plotted on a 2D graph, a linear fit was performed, and the obtained correlation efficient (Pearson’s R) was used to express the linearity and agreement between both datasets. The higher this factor, the higher the agreement between the two methods. For the Bland–Altman analysis, the difference between the measurement methods was calculated for each patient and plotted versus the average result of both methods. Further, by calculating the average and standard deviation of the measurement differences, one can observe the spread of the difference across the measurements and whether there is a bias of a measurement method.

## 3. Results

### 3.1. Patient Demographics

PBMC samples were collected from 15 patients with advanced high-grade serious ovarian cancer (HGSOC). Patient characteristics are displayed in Table 1.

### 3.2. Correlation of PD1 Populations between FACSymphony and the Chip

**Figure 3:**
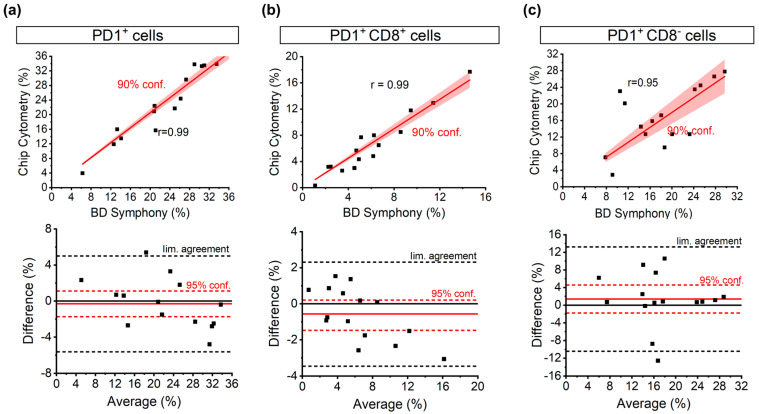
Pearson correlation and Bland–Altman agreement analysis for (**a**) total PD1+ cells, (**b**) CD8+PD1+ cells, and (**c**) CD8−PD1+ cells show high correlation and good agreement between chip cytometry and conventional flow cytometry in all three populations.

Both the general PD1+ population and the more defined CD8+PD1+ and CD8−PD1+ population relative to the total CD45+ population were determined. Correlation graphs of these populations measured via both methods show that the obtained data are scattered closely around the identity line, confirming that these two methods show a good similarity, with a high Pearson’s R value (0.99 for both PD1+ and CD8+PD1+ cells, 0.95 for CD8−PD1+ cells, Figure 3). The agreement between the two methods was further quantified using Bland–Altman plots in which the difference between the two measurement points is plotted versus the average. For the overall PD1+ population, the observed difference of the means is very close to equality (−0.31%) with a small negative bias for analysis on the chip. When the CD45+ population is subdivided into CD8+ and CD8− populations, the resulting difference of the means for both populations is higher but remains below 2% (−0.57% in CD8+ and 1.39% in CD8−).

### 3.3. Clinically Relevant Patterns Found on Conventional Cytometry Are Replicated through Chip Cytometry

**Figure 4:**
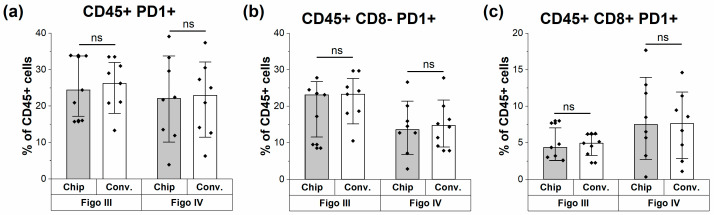
Clinical patterns of immune cells can be detected similarly on conventional and chip cytometry. (**a**) All CD45+ PD1+ cells as measured by flow and chip cytometry, respectively. No difference can be seen between the data obtained via both measurement methods. (**b**) Readout of CD8−PD1+ cells on flow and chip cytometer, respectively, shows the same impression of a non-significant higher mean of PD1 positivity in stage III (21.37% for conventional and 19.15% for chip flow cytometry) compared to stage IV (15.25% for conventional and 14.06% for chip flow cytometry). (**c**) Similar readout of CD8+PD1+ cells on flow and chip cytometer, respectively, after analysis. A two-tailed *t*-test was used to compare the datasets between both measurement methods (ns = *p* < 0.05).

Upon ovarian cancer diagnosis, patients are often classified as FIGO III or IV. In addition, PD1 populations are one of the key targets for therapy development [36]. Therefore, we focused on those two stages and assessed whether both methods are capable of capturing the same enumeration of PD1+ cells in the different subpopulations at both FIGO stages. Figure 4 shows that the chip flow cytometer was able to reproduce the PD1 enumeration found with conventional flow cytometry across the different FIGO stages. Consequently, the same patterns between the FIGO stages could be observed. When observing the relative abundance of CD45+ CD8−PD1+ cells, we observed a non-significant increase in FIGO stage III patients (Figure 4b; two-tailed *t*-test, *p* > 0.05). On the other hand, a non-significant decrease was seen in CD8+PD1+ cells at FIGO stage III (two-tailed *t*-test, *p* > 0.05). Although the limited number of patient samples does not allow us to draw a clinical conclusion, the observation that the exact same trend is visible using the chip flow cytometer validates this method for guiding both research and clinical diagnostics.

### 3.4. Higher Throughput Acquisition

**Figure 5:**
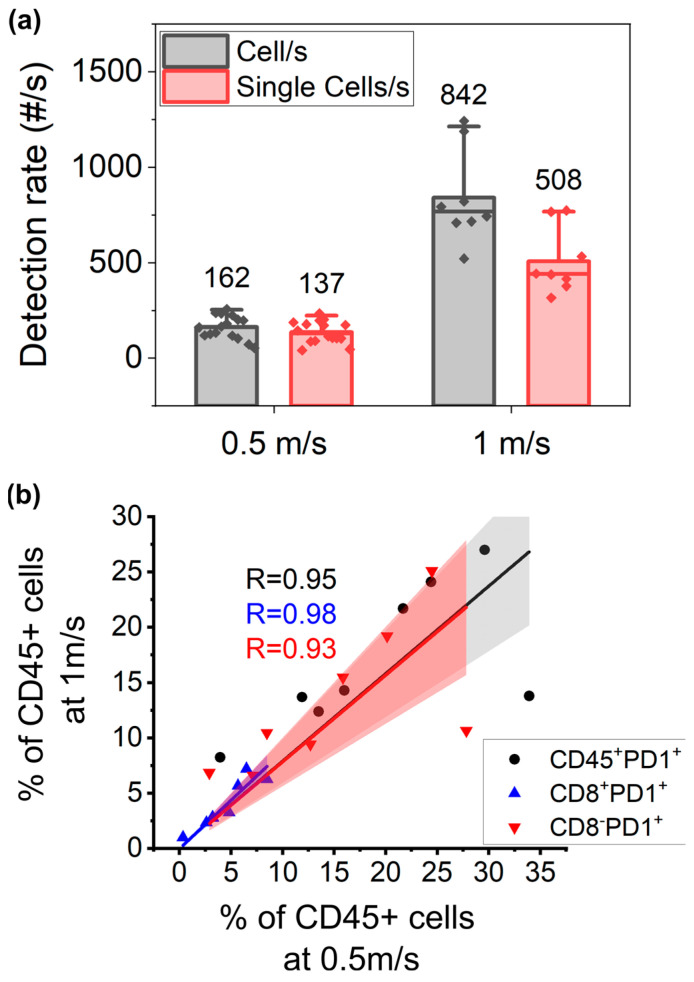
Higher speed acquisition does not hamper measurement sensitivity. (**a**) Comparison of total detections and single cells acquired at speeds of 0.5 m/s and 1 m/s shows that the chip can handle doublet discrimination at higher sample concentrations and (**b**) still generates the same fluorescence sensitivity compared to lower speed acquisition, as demonstrated by the Pearson correlation resulting from linear fit statistical analysis for total PD1+ cells (R = 0.94, black dots), CD8+PD1+ (R = 0.99, blue, full-colored triangle), and CD8−PD1+ (R = 0.92, red upside-down triangle) populations.

Although we envision parallelizing fluidic channels on the same silicon chip to increase the throughput, the flow speed and throughput of the single unit remains a relevant parameter to maximize the final throughput of the system. To assess whether the smaller integration time associated with higher flow speed would affect the sensitivity of the measurements, a fraction of eight measured samples was run on a chip at a higher speed of 1 m/s. It must be noted that in the condition of 1 m/s, the ratio of the sample to the sheath fluid was increased, similar to increasing the speed on a conventional cytometer. This allowed for a higher detection rate but also induced a higher occurrence of coincidence events which were excluded based on our gating strategy (Figure 5a, Supplementary Figure S2). The mean percentage of single cells relative to the total cells was significantly lower on the high-speed setting (59.87% ± 4.89) compared to the low-speed setting (83.49% ± 9.7; paired *t*-test, *p* = 0.000048). If we zoom in on the gated single-cell population, the measured PD1 subpopulations show a good correlation between both speed regimes, especially for the population exclusively defined by positive gating (the CD45+CD8+PD1+ population, which achieved a Pearson’s R correlation of 0.98) (Figure 5b). This demonstrates that a decreased integration time for fluorescence collection at this speed regime does not hamper the measurement sensitivity, including the detection of the weak PD1 marker. Further, it shows that the lower ratio of cell to sheath flow did not significantly affect the detection after proper doublet discrimination. Given that these chips can handle concentrations of 6 × 10^6^ cells/mL, as opposed to the concentrations currently used (1 × 10^6^ cells/mL), we envision that a detection rate of 5000 cells per second per cytometric channel can be achieved using this speed regime. Envisioning a parallelization of 20 channels per chip, we would be able to reach a throughput of 100,000 cells/s. This opens the possibility of developing multichannel chips that can analyze a larger volume of single cells with higher throughput than conventional flow cytometry instruments while maintaining high accuracy on the single-channel level.

## 4. Discussion

The ultimate goal of developing chip flow cytometry is to develop a point of care instrument enabling fast acquisition and readout of the immune profile in an outpatient setting without the need for highly trained staff. In addition, relevant clinical panels often rely on weak markers, such as PD-1, for recognition in a small subpopulation. This implies that you need a high throughput to achieve reliable measurement statistics while maintaining single-cell accuracy. The latter is especially compromised by merely increasing the throughput on a single channel, as in conventional flow cytometry. Therefore, we believe that the only way to further increase the throughput is by fluidic parallelization [34].

Aside from the obvious fluidic channel, this implies a need for optical multiplexing. To enable this, while keeping the overall device format suitable for an outpatient setting, we believe that a silicon platform allowing for fluidic and photonic integration is the best approach [19,20]. This implies that the fluidic concept should be easy to integrate and scale on a small footprint. We believe that only when these parameters are fulfilled, the added cost of the silicon platform will be outweighed by the advantages of high manufacturability and the addition of integrated extra features, such as optics, which will reduce the operational cost of the device [15]. For these purposes, we have designed our microfluidic unit cell based on the hydrodynamic focusing principle.

It is known that the step from microfluidic proof-of-concept demonstrations to actual biologically relevant samples derived from patient material causes technical issues, including clogging, viscosity variability, biomarker heterogeneity, and differences in background signals derived from patients versus healthy controls. This has made it extremely difficult to introduce microfluidics-based chip cytometry into a clinically relevant setting [13]. Therefore, in this proof-of-concept study, we show on a relevant clinical panel, that our microfluidics approach can perform immune fluorescent data acquisition with the same accuracy and sensitivity as conventional flow cytometry on a single channel.

First, the enumeration of the different cell types on the chip flow cytometer corresponded well to the conventional cytometer. The success in detecting dim PD1 marker expression with high sensitivity can be attributed to the absence of any autofluorescent signal using silicon channels sealed with a quartz cover, compared to other materials frequently used in microfluidics [37]. Secondly, when zooming in on the relevant FIGO stages apparent in clinical practice, we confirmed that the chip flow cytometer is capable of reproducing the data achieved on the conventional cytometer. As a consequence, same trends in the limited dataset could be seen and further explored using the flow chip cytometer in the future. Given the dim PD1 marker expression, these results show great promise for further development towards the creation of a compact chip cytometer for automated, clinical processing to enable faster decision making and therapeutic guidance. Our group, indeed, has already demonstrated that benign ovarian masses can be discriminated from malignant disease based on a peripheral immune panel [38]. However, it should be noted that the currently developed microfluidics chip flow cell used in this report was evaluated using bulk optical components. Although the flow cell itself has been optimized for the removal of any background signal, fluorescence detection sensitivity is still limited by the optical components used. Further optimization of this optical system and overall miniaturization still needs to be carried out.

The latter is associated with the second challenge in the development of a compact high-throughput cytometer: optical multiplexing which incorporates multiplexed illumination as well as fluorescence detection. At imec, we have developed a photonic platform for low-loss PECVD silicon nitride (SiN) waveguides for visible to near-infrared (NIR) (532–900 nm) wavelength applications [1,19,39]. Using this platform, we have shown that light can be guided to an interrogation point where fluorescent light is generated by the analyte or cell, collected, and routed to a detector [14,17]. In the field, it has been shown that these principles can be multiplexed to create multi-spot excitation patterns in a fluidically multiplexed chip [20].

**Figure 6:**
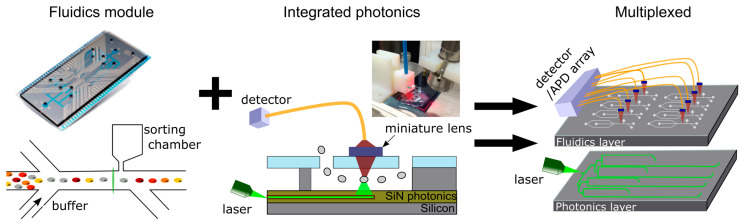
Concept of a multiplexed cytometer chip combining both fluidics and photonics module. The fluidics module harbors besides 2D hydrodynamic focusing also the capacity to sort [22]. The photonics module, presented in the middle, comprises an SiN waveguide platform enabling coupling of laser light, routing of light throughout the chip, and emission of light into the fluidic channel [14,17,19]. The fluorescence emission by the cells can be collected through a quartz top plate, focused to an optical fiber with miniature lenses, such as grin lenses, and collected by an APD or PMT detector. Both modules can be monolithically integrated and multiplexed as represented in the figure on the right.

However, to date, it remains uncertain whether the integrated system will allow for the same sensitivity required for the detection of clinically relevant weak markers in real-life samples [13]. A practical point of loss of light lies in the use of an optical fiber compared to an objective lens. However, this loss can be compensated by either the optimization of the downstream light path, preventing lossy dichroic mirrors, or by maximizing the incoupling of light into the optical fiber. Both advances have successfully led to high sensitivity in a new generation of conventional cytometers from Beckman Coulter [40]. The same concept could be explored on a chip by incorporating flat optics in the quartz top or in the silicon when all the collection is integrated [41]. Future work will focus on the integration of these modules in one monolithically integrated chip in a multiplexed format (Figure 6).

The envisioned throughput of the multiplexed cytometer chip can be estimated based on the current knowledge. With current optical components, we have shown that a detection speed of 1 m/s could be achieved, which would translate to 5000 cells/s using a sample concentration of only 6 × 10^6^/mL. Compared to the detection rate in current state-of-the-art flow cytometry [42], one could achieve 50,000 cells/s using 10 microfluidic channels. However, using the scalability inherent to both the photonics and microfluidics modules, we envision that more than 10 channels would be possible, competing with the throughput of the fastest flow cytometers available. Furthermore, using parallelization, which keeps the speed relatively low in a single channel, we can maintain a high accuracy on the single-cell level.

In the future, this platform could be even further developed to incorporate the full cell processing workflow, including staining, and washing steps. Current clinical workflows are a combination of automated single steps which are prone to human error. Adherence to specific laboratory protocols and in-depth knowledge of conventional flow cytometers is paramount to achieve reproducible and trustworthy clinical data [43]. Currently, processing at a central lab has been the standard way of working to reduce this variability. However, it has been shown that lyophilization and sample transport on its own induce variability, especially in low-abundance markers [44]. Although its development will entail a great endeavor, the availability of single steps on microfluidic chips supports the feasibility of combining these modules, using silicon technology, into one single device without the need for manual intervention and planning to transport the samples from one tool to another [45,46].

With this work, we have shown that detecting a biologically relevant, dim immune marker in a heterogenous group of ovarian cancer patients is feasible with a microfluidics-based chip cytometer; this type of cytometer can perform as effectively as a conventional flow cytometry. The next step is to demonstrate the real potential of chip cytometry in one integrated chip and expand the number of fluorescent detection channels to allow follow up of more complex immune panels.

On top of the integrated cytometry aspect, we believe we can reach the full clinical potential if downstream sorting would be integrated into this system, as demonstrated by De Wijs et al. [22]. For example, the sorting of tumor antigen-specific T cells with a high affinity could be used, not only to characterize patient cells in depth through T cell repertoire sequencing, but also for the development of patient-specific, adaptive cell (immuno)therapy, a highly promising treatment for cancer patients hampered—in part—by the absence of adequate cell-sorting techniques [47]. In addition to our current microfluidics-based chip cytometry design, numerous other devices and approaches have been reported based on mechanical, piezoelectric, dielectrophoretic, and acoustic principles. The achievable throughput is different for each actuation scheme, but our bubble jet technology looks most promising in view of higher throughputs, i.e., higher speeds up to 5 kHz and higher levels of parallelization [22]. This sorting performance fits well with the detection sensitivity observed in this study.

## 5. Conclusions

In conclusion, we have paved the way for the implementation of immune profiling in clinical practice. Our lab-on-chip fluidic concept and flow cell can produce the same immune results as a conventional flow cytometer device. The next steps in our development process include the integration of photonic illumination and collection and the parallelization of the shown concept.

## Figures and Tables

**Table 1:** Patient demographics of the 15 included patients with HGSOC.

Characteristic		Result *n* (%)
Age	Median (years), range	68 (49–76)
FIGO ^1^ stage at diagnosis	III	7 (47)
	IV	8 (53)
Timing of blood sampling	At diagnosis	4 (27)
	During primary treatment	8 (53) ^2^ *
	After recurrence	3 (20) ^2^ #
(Peg)filgrastim	Yes	5 (33)
	No	6 (40)
	Not applicable	4 (27)

Notes: ^1^ FIGO: International Federation of Gynecology and Obstetrics; ^2^ NACTIDS; ENGOT-OV43: SGNTV-002: A study of weekly tisotumab-vedotin for patients with platinum-resistant ovarian cancer with safety run-in (NCT03657043); * 5/8 at the moment of interval debulking surgery (IDS) after receiving neoadjuvant chemotherapy (NACT) (carboplatinum-paclitaxel); 3/8 at the moment of IDS after receiving NACT in the ENGOT-OV43 study (Study of chemotherapy with pembrolizumab followed by maintenance with olaparib for the first-line treatment of women with BRCA non-mutated advanced epithelial ovarian cancer (NCT03740165)); # 1/3 at recurrence diagnosis, 2/3 during treatment for recurrence.

## Data Availability

Raw data were generated at KU Leuven and imec. Derived data supporting the findings of this study are available from the corresponding author, L.L., on request.

## Supplementary Materials

The following supporting information can be downloaded at: https://www.mdpi.com/article/10.3390/cells13010007/s1, Figure S1: Gating strategy acquired on the chip cytometer, Figure S2: Gating strategy on a conventional cytometer, Table S1: Information on optical filters used in both systems, Table S2: Individual data per patient as recorded by BD FACSymphony and on chip cytometry setup.

## References

[1] Globocan. https://gco.iarc.fr/survival/survmark/visualizations/viz1/?groupby=%22cancer_site%22&period=%225%22&cancer_site=%22Colon%22&country=%22Australia%22&year=%222014%22&gender=%22Females%22&sorting=%222%22.

[2] Peres L.C., Cushing-Haugen K.L., Köbel M., Harris H.R., Berchuck A., Rossing M.A., Schildkraut J.M., Doherty J.A. (2019). Invasive Epithelial Ovarian Cancer Survival by Histotype and Disease Stage. J. Natl. Cancer Inst..

[3] SEER Cancer Statistics. https://seer.cancer.gov/statfacts/html/ovary.html.

[4] Schadendorf D., Hodi F.S., Robert C., Weber J.S., Margolin K., Hamid O., Patt D., Chen T.T., Berman D.M., Wolchok J.D. (2015). Pooled Analysis of Long-Term Survival Data from Phase II and Phase III Trials of Ipilimumab in Unresectable or Metastatic Melanoma. J. Clin. Oncol..

[5] Larkin J., Chiarion-Sileni V., Gonzalez R., Grob J.-J., Rutkowski P., Lao C.D., Cowey C.L., Schadendorf D., Wagstaff J., Dummer R. (2019). Five-Year Survival with Combined Nivolumab and Ipilimumab in Advanced Melanoma. N. Engl. J. Med..

[6] Disis M.L., Taylor M.H., Kelly K., Beck J.T., Gordon M., Moore K.M., Patel M.R., Chaves J., Park H., Mita A.C. (2019). Efficacy and Safety of Avelumab for Patients with Recurrent or Refractory Ovarian Cancer: Phase 1b Results from the JAVELIN Solid Tumor Trial. JAMA Oncol..

[7] Heindl A., Lan C., Rodrigues D.N., Koelble K., Yuan Y. (2016). Similarity and Diversity of the Tumor Microenvironment in Multiple Metastases: Critical Implications for Overall and Progression-Free Survival of High-Grade Serous Ovarian Cancer. Oncotarget.

[8] Jiménez-Sánchez A., Memon D., Pourpe S., Veeraraghavan H., Li Y., Vargas H.A., Gill M.B., Park K.J., Zivanovic O., Konner J. (2017). Heterogeneous Tumor-Immune Microenvironments among Differentially Growing Metastases in an Ovarian Cancer Patient. Cell.

[9] Zhang A.W., McPherson A., Milne K., Kroeger D.R., Hamilton P.T., Miranda A., Funnell T., Little N., de Souza C.P.E., Laan S. (2018). Interfaces of Malignant and Immunologic Clonal Dynamics in Ovarian Cancer. Cell.

[10] de Bruyn C., Ceusters J., Landolfo C., Baert T., Thirion G., Claes S., Vankerckhoven A., Wouters R., Schols D., Timmerman D. (2021). Neo-Adjuvant Chemotherapy Reduces, and Surgery Increases Immunosuppression in First-Line Treatment for Ovarian Cancer. Cancers.

[11] Park Y.H., Lal S., Lee J.E., Choi Y.-L., Wen J., Ram S., Ding Y., Lee S.H., Powell E., Lee S.K. (2020). Chemotherapy Induces Dynamic Immune Responses in Breast Cancers That Impact Treatment Outcome. Nat. Commun..

[12] Chen W., Huang N.T., Li X., Yu Z.T.F., Kurabayashi K., Fu J. (2013). Emerging Microfluidic Tools for Functional Cellular Immunophenotyping: A New Potential Paradigm for Immune Status Characterization. Front. Oncol..

[13] Yang R.J., Fu L.M., Hou H.H. (2018). Review and Perspectives on Microfluidic Flow Cytometers. Sens. Actuators B Chem..

[14] Verellen N., Vercruysse D., Rochus V., Du Bois B., Dusa A., Kerman S., Mahmud-Ul-Hasan M., Van Dorpe P., Rottenberg X., Lagae L. Integrated Photonics for Miniature Flow Cytometry. Proceedings of the 49th International Conferece on Solid State Devices and Materials—SSDM.

[15] Zhang Y., Watts B.R., Guo T., Zhang Z., Xu C., Fang Q. (2016). Optofluidic Device Based Microflow Cytometers for Particle/Cell Detection: A Review. Micromachines.

[16] Friis P., Hoppe K., Leistiko O., Mogensen K.B., Rg Hü Bner J., Rg J., Kutter P. (2001). Monolithic Integration of Microfluidic Channels and Optical Waveguides in Silica on Silicon. Appl. Opt..

[17] Kerman S., Vercruysse D., Claes T., Stassen A., Mahmud Ul Hasan M., Neutens P., Mukund V., Verellen N., Rottenberg X., Lagae L. (2017). Integrated Nanophotonic Excitation and Detection of Fluorescent Microparticles. ACS Photon..

[18] Rosenauer M., Vellekoop M.J. (2010). Characterization of a Microflow Cytometer with an Integrated Three-Dimensional Optofluidic Lens System. Biomicrofluidics.

[19] Porcel M.A.G., Hinojosa A., Jans H., Stassen A., Goyvaerts J., Geuzebroek D., Geiselmann M., Dominguez C., Artundo I. (2019). Silicon Nitride Photonic Integration for Visible Light Applications. Opt. Laser Technol..

[20] Ozcelik D., Jain A., Stambaugh A., Stott M.A., Parks J.W., Hawkins A., Schmidt H. (2017). Scalable Spatial-Spectral Multiplexing of Single-Virus Detection Using Multimode Interference Waveguides. Sci. Rep..

[21] Dumitru A., Dobrica E.C., Croitoru A., Cretoiu S.M., Gaspar B.S. (2022). Focus on PD-1/PD-L1 as a Therapeutic Target in Ovarian Cancer. Int. J. Mol. Sci..

[22] De Wijs K., Liu C., Dusa A., Vercruysse D., Majeed B., Tezcan D.S., Blaszkiewicz K., Loo J., Lagae L. (2017). Micro Vapor Bubble Jet Flow for Safe and High-Rate Fluorescence-Activated Cell Sorting. Lab Chip.

[23] Kung Y.C., Huang K.W., Chong W., Chiou P.Y. (2016). Microfluidics: Tunnel Dielectrophoresis for Tunable, Single-Stream Cell Focusing in Physiological Buffers in High-Speed Microfluidic Flows (Small 32/2016). Small.

[24] Yu C., Vykoukal J., Vykoukal D.M., Schwartz J.A., Shi L., Gascoyne P.R.C. (2005). A Three-Dimensional Dielectrophoretic Particle Focusing Channel for Microcytometry Applications. J. Microelectromech. Syst..

[25] Yan S., Zhang J., Li M., Alici G., Du H., Sluyter R., Li W. (2014). On-Chip High-Throughput Manipulation of Particles in a Dielectrophoresis- Active Hydrophoretic Focuser. Sci. Rep..

[26] Gao Y., Wu M., Lin Y., Xu J. (2020). Acoustic Microfluidic Separation Techniques and Bioapplications: A Review. Micromachines.

[27] Zalis M.C., Reyes J.F., Augustsson P., Holmqvist S., Roybon L., Laurell T., Deierborg T. (2016). Label-Free Concentration of Viable Neurons, HESCs and Cancer Cells by Means of Acoustophoresis. Integr. Biol..

[28] Dykes J., Lenshof A., Åstrand-Grundström I.B., Laurell T., Scheding S. (2011). Efficient Removal of Platelets from Peripheral Blood Progenitor Cell Products Using a Novel Micro-Chip Based Acoustophoretic Platform. PLoS ONE.

[29] Dao M., Suresh S., Huang T.J., Li P., Mao Z., Peng Z., Zhou L., Chen Y., Huang P.H., Truica C.I. (2015). Acoustic Separation of Circulating Tumor Cells. Proc. Natl. Acad. Sci. USA.

[30] Zmijan R., Jonnalagadda U.S., Carugo D., Kochi Y., Lemm E., Packham G., Hill M., Glynne-Jones P. (2015). High Throughput Imaging Cytometer with Acoustic Focussing. RSC Adv..

[31] Piyasena M.E., Suthanthiraraj P.P.A., Applegate R.W., Goumas A.M., Woods T.A., López G.P., Graves S.W. (2012). Multinode Acoustic Focusing for Parallel Flow Cytometry. Anal. Chem..

[32] Pritchard R.H., Zhukov A.A., Fullerton J.N., Want A.J., Hussain F., la Cour M.F., Bashtanov M.E., Gold R.D., Hailes A., Banham-Hall E. (2019). Cell Sorting Actuated by a Microfluidic Inertial Vortex. Lab Chip.

[33] Hulspas R., Villa-Komaroff L., Koksal E., Etienne K., Rogers P., Tuttle M., Korsgren O., Sharpe J.C., Berglund D. (2014). Purification of Regulatory T Cells with the Use of a Fully Enclosed High-Speed Microfluidic System. Cytotherapy.

[34] Zhukov A.A., Pritchard R.H., Withers M.J., Hailes T., Gold R.D., Hayes C., la Cour M.F., Hussein F., Rogers S.S. (2021). Extremely High-Throughput Parallel Microfluidic Vortex-Actuated Cell Sorting. Micromachines.

[35] Wang C., Ma Y., Chen Z., Wu Y., Song F., Qiu J., Shi M., Wu X. (2021). Sheatless Microflow Cytometer Utilizing Two Bulk Standing Acoustic Waves. Cytometry.

[36] Pawłowska A., Suszczyk D., Tarkowski R., Paduch R., Kotarski J., Wertel I. (2020). Programmed Death-1 Receptor (PD-1) as a Potential Prognosis Biomarker for Ovarian Cancer Patients. Cancer Manag. Res..

[37] Piruska A., Nikcevic I., Lee S.H., Ahn C., Heineman W.R., Limbach P.A., Seliskar C.J. (2005). The Autofluorescence of Plastic Materials and Chips Measured under Laser Irradiation. Lab Chip.

[38] Coosemans A., Baert T., Ceusters J., Busschaert P., Landolfo C., Verschuere T., van Rompuy A.S., Vanderstichele A., Froyman W., Neven P. (2019). Myeloid-Derived Suppressor Cells at Diagnosis May Discriminate between Benign and Malignant Ovarian Tumors. Int. J. Gynecol. Cancer.

[39] Subramanian A.Z., Neutens P., Dhakal A., Jansen R., Claes T., Rottenberg X., Peyskens F., Selvaraja S., Helin P., Dubois B. (2013). Low-Loss Singlemode PECVD Silicon Nitride Photonic Wire Waveguides for 532-900 Nm Wavelength Window Fabricated within a CMOS Pilot Line. IEEE Photon. J..

[40] Butement J.T., Horak P., Wilkinson J.S., Butement J.T., Holloway P.M., Welsh J.A., Holloway J.A., Englyst N.A., West J., Holloway P.M. (2020). Monolithically-Integrated Cytometer for Measuring Particle Diameter in the Extracellular Vesicle Size Range Using Multi-Angle Scattering. Lab Chip.

[41] Mangal N., Snyder B., Van Campenhout J., Van Steenberge G., Missinne J. (2021). Monolithic Integration of Microlenses on the Backside of a Silicon Photonics Chip for Expanded Beam Coupling. Opt. Express.

[42] Picot J., Guerin C.L., Le Van Kim C., Boulanger C.M. (2012). Flow Cytometry: Retrospective, Fundamentals and Recent Instrumentation. Cytotechnology.

[43] Glier H., Heijnen I., Hauwel M., Dirks J., Quarroz S., Lehmann T., Rovo A., Arn K., Matthes T., Hogan C. (2019). Standardization of 8-Color Flow Cytometry across Different Flow Cytometer Instruments: A Feasibility Study in Clinical Laboratories in Switzerland. J. Immunol. Methods.

[44] Ivison S., Malek M., Garcia R.V., Broady R., Halpin A., Richaud M., Brant R.F., Wang S.I., Goupil M., Guan Q. (2018). A Standardized Immune Phenotyping and Automated Data Analysis Platform for Multicenter Biomarker Studies. JCI Insight.

[45] Patibandla P.K., Estrada R., Kannan M., Sethu P. (2014). A Microfluidics-Based Technique for Automated and Rapid Labeling of Cells for Flow Cytometry. J. Micromech. Microeng..

[46] Kim B., Kim K.H., Chang Y., Shin S., Shin E.C., Choi S. (2019). One-Step Microfluidic Purification of White Blood Cells from Whole Blood for Immunophenotyping. Anal. Chem..

[47] Morotti M., Albukhari A., Alsaadi A., Artibani M., Brenton J.D., Curbishley S.M., Dong T., Dustin M.L., Hu Z., McGranahan N. (2021). Promises and Challenges of Adoptive T-Cell Therapies for Solid Tumours. Br. J. Cancer.

