# Supplementary material for: A Microfluidics Approach for Ovarian Cancer Immune Monitoring in an Outpatient Setting
Source: Cells. 2023 Dec 20;13(1):7. doi: 10.3390/cells13010007 (PMC10778191; doi:10.3390/cells13010007)
Supplement: Supplementary file 1 [file cells-13-00007-s001.zip › cells-2711963-supplementary.pdf]

Supplementary information

Supplementary Table S1. Information on optical filters used in both systems. In the BD FACSymphony, a 651 nm yellow-green laser was used while on the Chip cytometry setup, the optical path is based on a 532 nm green laser.

Fluorophore	BD FACSymphony		Chip cytometry setup	
	LP filter	BP Filter	LP filter	BP filter
PE	/	586/15	/	572/28
PE-Dazzle	600	610/20	593	615/20
PE-Cy5	635	670/30	635	675/67
PE-Cy7	750	780/60	740	785/62

Supplementary Table S2. Individual data per patient as recorded by BD FACSymphony and on chip cytometry setup. Total number of recorded $CD45^+$ cells are given, in addition to percentages of the total $CD45^+$ population respective populations.

	BD FACSymphony				Chip cytometer			
	absolute cell count	percentage from CD45+ cells			absolute cell count	percentage from CD45+ cells		
Patient count	CD45+	CD45+ PD1+	CD45+ CD8+ PD1+	CD45+ CD8- PD1+	CD45+	CD45+ PD1+	CD45+ CD8+ PD1+	CD45+ CD8- PD1+
1	130737	20,9	11,4232	14,3172	66161	22,4	12,9352	14,5024
2	112849	25	8,5796	16,3944	32883	21,7	8,4942	15,8562
3	77064	12,6	4,704	7,82144	37970	11,9	5,675	7,1116
4	86969	21,1	4,5356	18,6524	28577	15,7	3,003	9,487
5	58837	33,5	6,1815	29,69225	507554	33,9	4,81164	27,80212
6	64535	26,2	3,4602	25,25508	26661	24,4	2,60307	24,46521
7	81104	20,8	4,9236	18,0544	47876	20,9	4,344	17,248
8	67803	6,27	1,10097	9,0949	47096	3,94	0,334908	2,889138
9	78491	31	6,2466	24,2018	29641	33,5	7,995	23,506
10	84055	30,5	14,6142	15,1774	40497	33,3	17,6788	12,6828
11	79259	37,4	9,4563	27,7715	60486	39,1	11,7943	26,6198
12	68849	27,3	6,6591	20,0455	86098	29,6	6,4872	20,1312
13	87501	14,1	2,46257	11,38138	62523	13,5	3,21993	12,68634
14	306322	29	5,1135	23,27	65999	33,8	7,6934	23,0533
15	100985	13,3	2,268	10,492	30647	16	3,192	8,4864

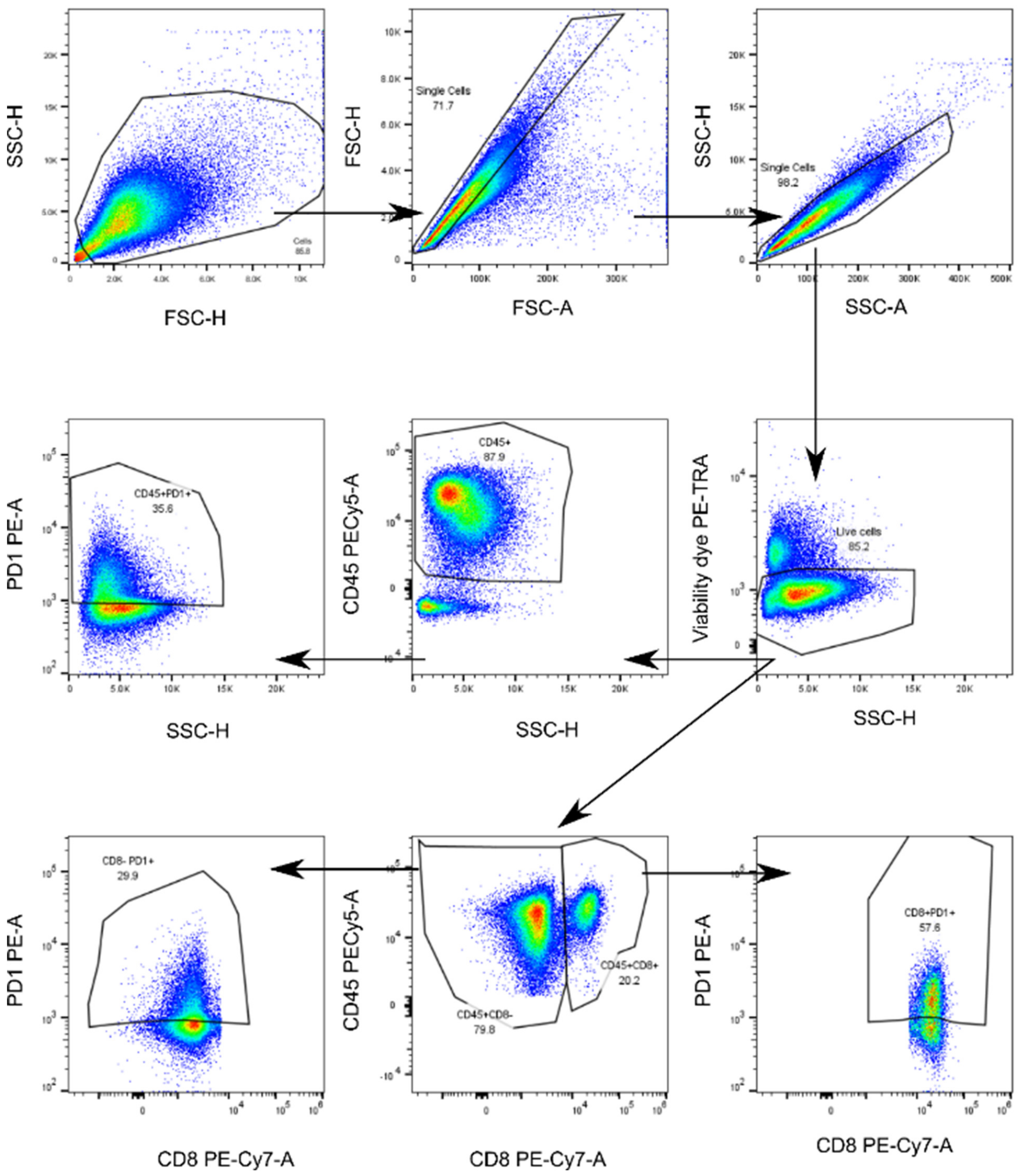

Supplementary Figure S1. Gating strategy on the chip cytometer. The data processing of a representative sample is indicated step by step.

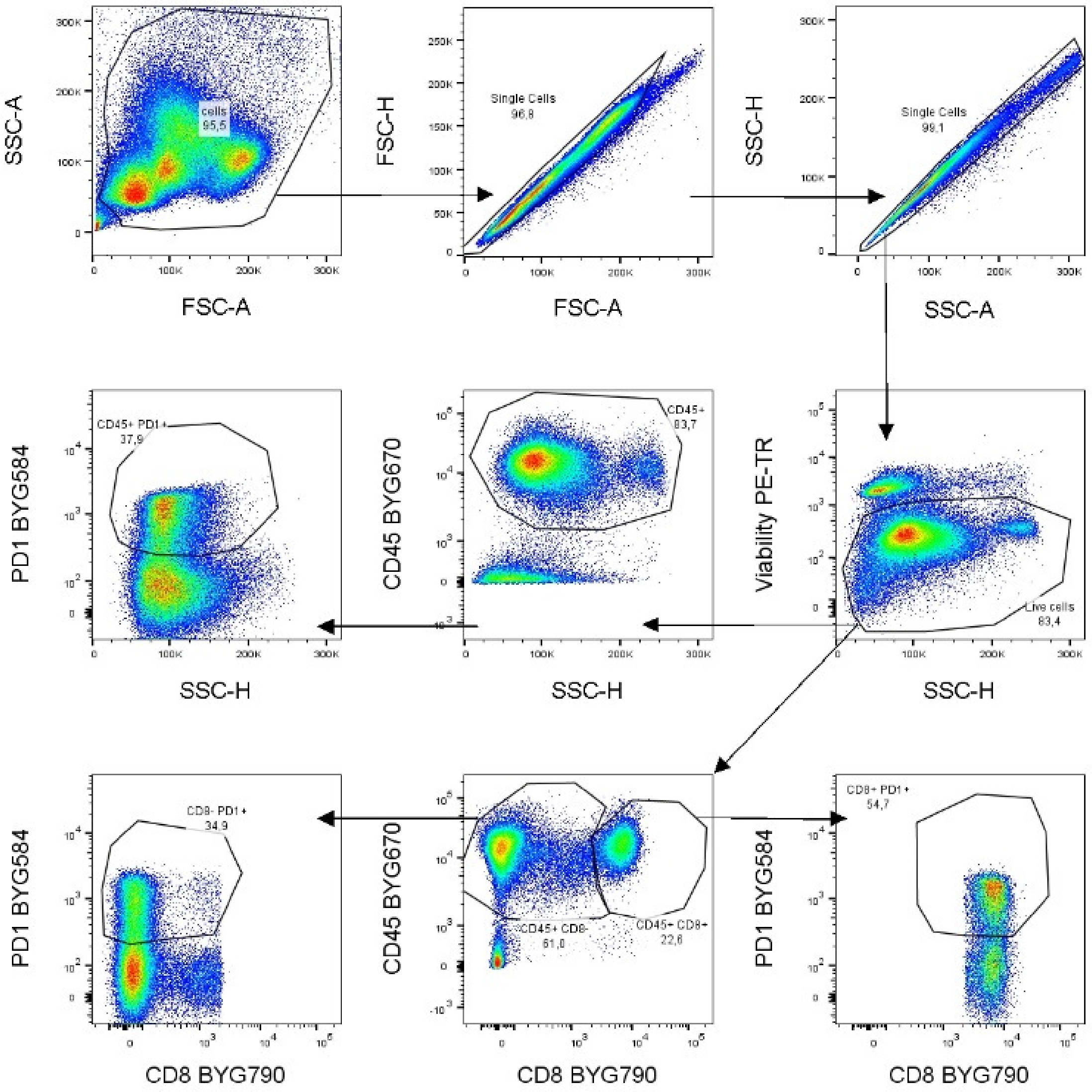

Supplementary Figure S2. Gating strategy on a conventional flow cytometer. The stepwize analysis proces of the same sample as illustrated in Supplementary Figure 1 is respresented.
